# Robust Indoor Human Activity Recognition Using Wireless Signals

**DOI:** 10.3390/s150717195

**Published:** 2015-07-15

**Authors:** Yi Wang, Xinli Jiang, Rongyu Cao, Xiyang Wang

**Affiliations:** School of Software, Dalian University of Technology, Dalian 116620, China; E-Mails: jiangxl@dlut.edu.cn (X.J.); caocao7066@outlook.com (R.C.); ssdutwxy@foxmail.com (X.W.)

**Keywords:** wireless sensing, channel state information, action recognition

## Abstract

Wireless signals–based activity detection and recognition technology may be complementary to the existing vision-based methods, especially under the circumstance of occlusions, viewpoint change, complex background, lighting condition change, and so on. This paper explores the properties of the channel state information (CSI) of Wi-Fi signals, and presents a robust indoor daily human activity recognition framework with only one pair of transmission points (TP) and access points (AP). First of all, some indoor human actions are selected as primitive actions forming a training set. Then, an online filtering method is designed to make actions’ CSI curves smooth and allow them to contain enough pattern information. Each primitive action pattern can be segmented from the outliers of its multi-input multi-output (MIMO) signals by a proposed segmentation method. Lastly, in online activities recognition, by selecting proper features and Support Vector Machine (SVM) based multi-classification, activities constituted by primitive actions can be recognized insensitive to the locations, orientations, and speeds.

## 1. Introduction

Vision-based human activity analysis attempts to understand the movements of the human body using computer vision and machine learning techniques. Many studies have been done in recent years [1,2,3,4,5], however, robust action recognition is still a challenging problem due to the following issues: (a) Body parts or big size obstacles may cause partial occlusions; (b) An action, observed from different viewpoints, has different appearances; (c) Clothing, especially long skirts, may lead to apparent anthropometric differences; (d) The start-time and end-time points of an action are sometimes hard to detect accurately; (e) Dynamic backgrounds may make it difficult to locate and observe actions; (f) Smoke-filled, dim, or dark rooms may make it hard to observe actions; (g) People may feel uncomfortable with a camera overhead, especially in a bathroom. To tackle any one of these problems is an arduous task, and they are likely to appear at the same time in practical applications.

Recent advances in the wireless community give solutions for the above problems in a new way [6,7,8,9]. Studies [10,11] have proved that the existence and movement of humans will affect the channel state information (CSI) of wireless signals, and CSI has an advantage over light, infrared, or thermal energy when attempting to infer people’s movements. CSI holds potential for the convergence of accurate and pervasive indoor localization and has attracted numerous recent research efforts [10,11,12,13,14]. Studies [10,11,12] have shown that different actions have different CSI change patterns. When an individual conducts out-of-place actions and some in-place actions such as a fall, CSI values have great amplitude variance. But some in situ local body actions, such as playing on the computer, watching TV, eating food, cooking, and bathing, cannot cause obvious fluctuations of CSIs. Wi-See [12] is the first wireless system that can identify nine gestures in line-of-sight, non-line-of-sight, and through-the-wall scenarios. E-eyes [13] can distinguish a set of in-place human activities with only a single Wi-Fi access point. Paper [14] can accurately classify five states of the shopper during a typical in-store visit.

In this paper, we try to make a further step in solving the above vision-based issues for robust indoor, full-body action recognition by exploring the properties of CSI of Wi-Fi Wireless multi-input multi-output (MIMO) radios. To be specific:

(1) A framework for recognizing indoor human actions is proposed based on the recognition of the combination of primitive actions. Some indoor human actions are selected as primitive motions forming a training set at first. Then, in online recognition, a coarse detection is used to distinguish in-place activities from walking to continuous movement.
(2) A new signal preprocess and segmentation method is presented by exploring the properties of CSIs of Wi-Fi signals. An online filtering method is designed to let actions’ CSIs value curves be smooth and contain enough pattern information. And each primitive action’s pattern can be segmented from the outliers of CSIs accurately.
(3) By Kernel SVM based multi-classification with a feature selection method, many activities from the combination of primitive actions can be recognized efficiently insensitive to the location, orientation, speed, and anthropometric differences.

## 2. Background

### 2.1. Channel State Information (CSI)

CSI refers to channel properties in wireless communications [15]. CSI describes how a signal propagates from the transmitter to the receiver, and reveals a set of channel measurements depicting the amplitudes and phases of every subcarrier (see Equation (1)).

(1) $$H\left(f_{k}\right)=\left|\left|H\left(f_{k}\right)\right|\right|e^{jsin\left(∠H\right)}$$

where $H\left(f_{k}\right)$ is the CSI value at the subcarrier with central frequency of $f_{k}$ , and ∠H is the phase. In general, the receiver evaluates and quantitates CSI, then makes feedback to the sender (a time-division duplex system often needs reverse evaluation). In real application, CSI can be divided into instantaneous CSI and statistical CSI.

### 2.2. The Free Space Propagation Model

The free space propagation model assumes the ideal propagation condition that there is only one clear line-of-sight path between the transmitter and receiver. The received signal power in free space (usually air) at distance *d* from the transmitter with no obstacles nearby can be calculated by:

(2) $$P_{r}\left(d\right)=\frac{P_{t}G_{t}G_{r}\lambda^{2}}{{\left(4\pi \right)}^{2}d^{2}L}$$

where *P_t_* is the transmitted signal power. *G_t_* and *G_r_* are the antenna gains of the transmitter and the receiver, respectively. *L* (L ≥ 1) is the system loss, and λ is the wavelength.

The two-ray ground reflection model considers both the direct path and a ground reflection path. It is shown that this model gives more accurate prediction at a long distance than the free space model. The received power at distance d is predicted by Equation (3) [15]:

(3) $$P_{r}\left(d\right)=\frac{P_{t}G_{t}G_{r}h_{t}^{2}h_{r}^{2}}{d^{4}L}$$

where *h_t_* and *h_r_* are the heights of the transmitter’s antenna and receiver’s antenna, respectively. However, this two-ray model does not give a good result for short distance due to the oscillation caused by the constructive and destructive combination of the two rays. Instead, the free space model is still used when *d* is small. Therefore, when *d ≤ d_c_*, Equation (2) is used. Otherwise Equation (3) is used. So *d_c_* can be calculated as $d_{c}=\left(4\pi h_{t}h_{r}\right)/\lambda$ .

## 3. Methodology

### 3.1. Preparation

Leveraging the off-the-shelf Intel 5300 Network Interface Cards (NICs) and a modified driver, a group of sampled versions of Channel Frequency Responses (CFRs)—within the Wi-Fi bandwidth are revealed to upper layers in the format of the CSI. Especially as an open CSI Tool [16] built on the Intel Wi-Fi Wireless Link 5300 802.11n, MIMO radios with open source Linux wireless drivers are available and various applications have been performed [11,17,18]. So, in our work, we use this tool to gather CSIs.

**Figure 1:**
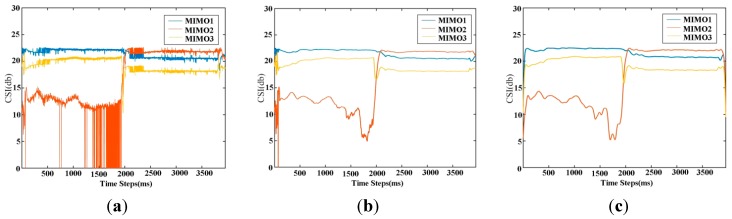
The MIMO subplots of sitting down onto a chair. (**a**) The original CSIs; (**b**) The filtered CSIs by average weight; (**c**) The filtered CSIs by Single-sideband Gaussian (SG) weight. It is obvious SG weight outperforms the average weight in time region 0–5 ms and 1500–2000 ms.

An *Ntx* × *Nrx* × 30 matrix is taken as the data structure of the CSI, where the third dimension is across 30 subcarriers in the Orthogonal Frequency Division Multiplexing (OFDM) channel. In an Intel 5300 NIC, there is only one transmitting terminal and three receiving ends, so it is a 1 × 3 MIMO system. We aggregate 30 subcarriers’ CSI values into one single value by their average for each MIMO plot, e.g., Figure 1a.

### 3.2. Filtering

Original CSIs fluctuate greatly due to environmental factors, so the statistical CSI is often adopted in real applications [11,12]. It is worth noting that a good online filtering algorithm to compute statistical CSIs is a fundamental basis for the following feature extraction and action recognition steps. Paper [11] uses a weight average value *w* from time steps *t* *−* 1 *−* *m* to *t* *−* 1 as the CSI value of time *t*, e.g., Figure 1b. Since its weight is decreasing in a linear fashion, the filtered signals still have jagged edges with a small *m*. On the contrary, if *m* is large, the signals are too smooth and may lose transmitting patterns. In contrast, we take a Single-sideband Gaussian (SG) kernel function in Equation (4) as the weight by executing a convolutional computation to get *CSI_t_*. Results demonstrate that (e.g., in Figure 1) SG weight has better smooth and shape-preserving ability for the original signals compared with [11] the same neighbor size.

(4) $$CSI_{t}=\;{ae}^{\frac{-{\left(x-b\right)}^{2}}{2c^{2}}}\times \left\{CSI_{t-1},CSI_{t-2},\ldots ,CSI_{t-1-m}\right\},\;\;\left(x\leq b\right)$$

### 3.3. Pattern Segmentation

Effective and accurate segmentation for an action from the signal sequence is the major premise of feature extraction and recognition. In wireless communication systems, the receiver computes the average received energy over a small duration to detect the start-time and end-time points of a packet [12]. When it comes to action recognition, it is more demanding. There are many anomaly detection methods that can be used here, especially the density-based technique, which is more suitable for the MIMO curves [19,20,21]. An instance that lies in a neighborhood with low density is declared to be an outlier. The local density is estimated by a specific distance at which a point can be reached from its neighbors [20]. The local outlier factor (LOF) can be valued by the ratio of average local densities of one instance’s neighbors (in time steps (*t* − *m*, …, *t* − 1)) to the local density of the instance (in time step *t*) [20,21]. In the following, we will present our pattern segmentation method in offline and online manners.

(1) Offline Pattern Segmentation

**Figure 2:**
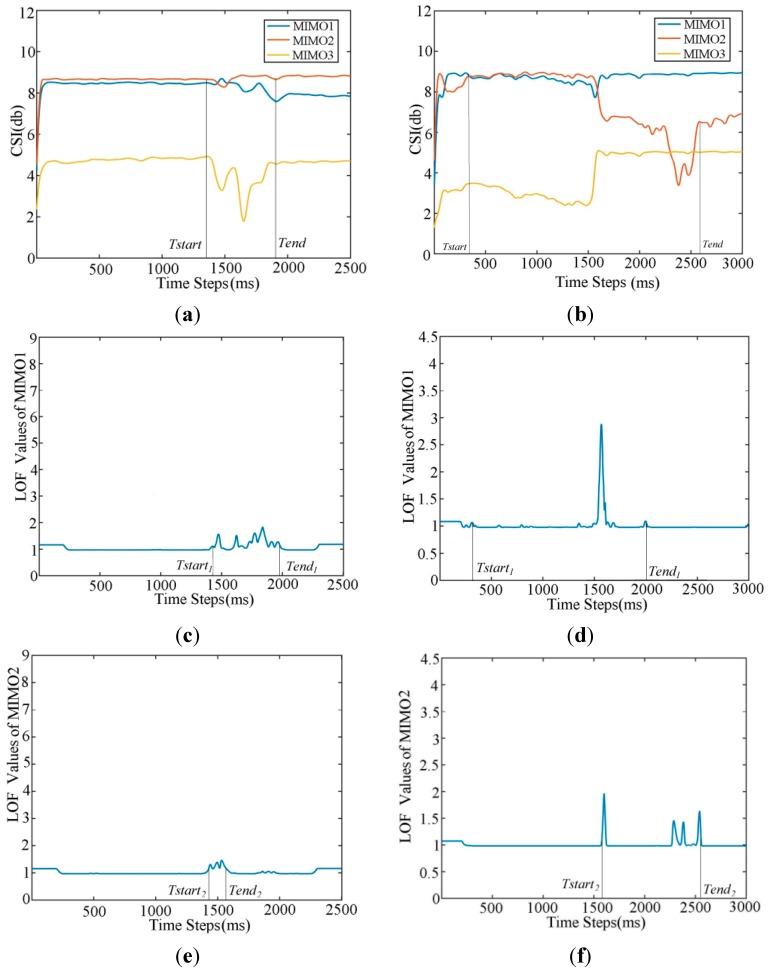

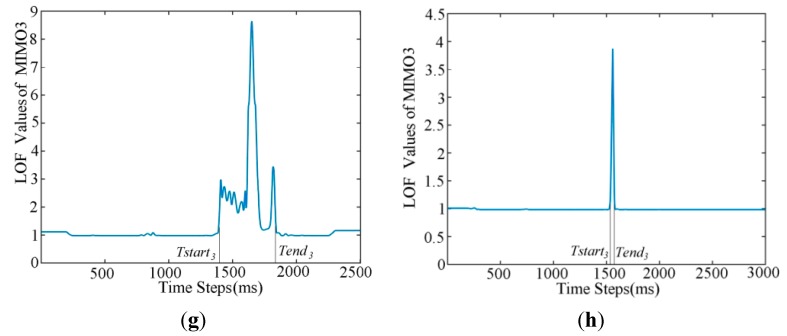
An example of action boundary computation by the LOFs of MIMOs: (**a**,**c**,**e**,**g**) Action 1; (**b**,**d**,**f**,**h**) Action 2; (**a**,**b**) Two actions’ MIMO subplots with their boundaries denoted by the vertical bars; (**c**–**h**) Two actions’ LOF subplots with their boundaries denoted by the vertical bars.

After computing the outliers for every MIMO subplot, we get outlier curves, e.g., in Figure 2c–h, and the next step is to determine the boundary of an action.

Though we have used the SG filter to smooth signals and ensure the environment is free from interference before and after an action (with only one action in the subplots) of training data, there still exist some false outliers; we should filter out the small outliers of small values. As we can see from Figure 2c–h, an outlier curve often presents a normal distribution in the region of the local peak, and some actions have a relatively static duration between two peaks. Therefore, we make the first time point whose gradient’s absolute value is larger than the threshold ε the boundary start point *Tstart_i_* and the last one whose gradient’s absolute value is larger than ε as boundary end point *Tend_i_* for MIMO_*i*_ (*i* = 1, …, 3) accordingly.

There is still another issue that should be considered, which is the correlation of three MIMOs. Most studies only use only one MIMO, which is the most fluctuated, to extract action patterns. However, the MIMOs received by the other two antennas do have some useful information about an action, as seen in the example shown in Figure 2. Consequently, we segment three MIMOs by combining the their boundaries to get a global boundary: {*Tstart* = *Min*{*Tstart_i_*}, *Tend* = *Max*{*Tend_i_*} (*i* = 1, …, 3)}. Using a stack data structure, this method can identify the boundary in an online manner. Then, we store the three segments of the original three MIMOs into a matrix as the results. Here, we do not directly use the LOF segments as an action pattern source as the work [11] did, for they are sometimes too sensitive to the fluctuations of CSIs. However, some properties of the local peak point of LOF curves may be beneficial for the discrimination of some action patterns, so we will use them in the feature selection steps in Section 3.4.

(2) Online Segmentation

**Figure 3:**
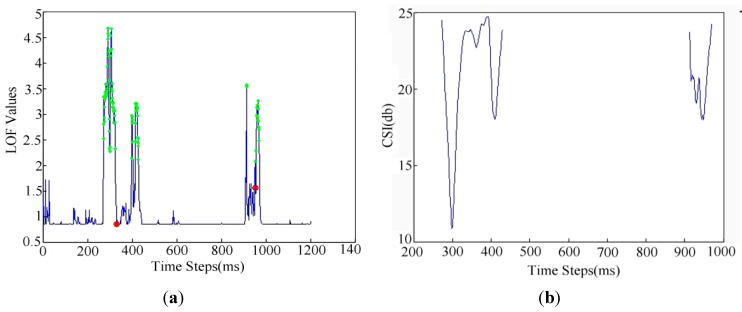
An example of online segmentation for two actions. (**a**) Two actions’ LOF subplots; (**b**) The segmentation result for two actions’ CSIs subplots by our method.

CSIs are similar to speech signals, which can be classified into three states: the silence state (SS), the transitional state (TS), and the action state (AS). An action’s CSIs will normally go under the five states of SS–TS–AS–TS–SS. Due to the mechanism of human bodies, their actions normally have time intervals. So, we can use a time threshold to segment in-place actions roughly. Then we use the K-Means [1] to get the centers of actions from anomalous points of CSIs, e.g., in Figure 3, where there are two actions and by K-means we can get two clusters. Then by the proposed pattern segmentation method, we can get each action’s pattern data. Finally, in order to distinguish walking activities and in-place activities, we can adopt a cumulative moving variance of CSIs with a threshold.

### 3.4. Feature Extraction

From the equations in Section 2, we can infer that the speed, orientation, and location of performing an action are the three major interference factors for action recognition based on wireless signals.

In a wireless system, different users performing the actions at different speeds only changes the duration of each segment, but does not change the pattern of positive and negative shifts [10] and the actions performed at different speeds may result in a similar pattern accordingly. As to the varieties of the same action in time and strength, dynamic time warping (DTW) [22] can be adopted to find an optimal alignment between two given (time-dependent) CSI sequences under restrictions.

**Figure 4:**
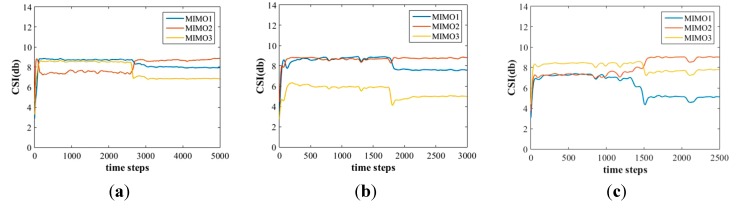
The MIMOs subplots for an action (e.g., squatting down to pick up something from the standing state) performed in three different orientations (**a**–**c**).

Performing the same action in different orientations will cause the reflection area to be varied. However, using the MIMO mechanism, this affect is not very obvious; e.g., in Figure 4, where a tester performed the same action in three directions: facing the transmission point (TP), perpendicular to the line-of-sight, and facing the access point (AP). The action patterns are similar, except for the relationship of the MIMOs, so we tried to avoid putting this into the features by generating the features of three MIMOs respectively.

CSIs in different locations have been explored by many studies to locate people in a room and have had positive results [10,14,18]. However, we want the features in our method to be insensitive to the locations.

Above all, we chose six statistic data of each MIMO subplot, and they are: (1) the normalized standard deviation (NSD); (2) the period of an action (PA); (3) the signal entropy (SE); (4) the interquartile range (IR); (5) the median absolute deviation (MAD); and (6) the range (Rg). Then we chose the total number of local max outliers (LMON) and the maximum value of outliers (MaxOV) of the LOF curves of each MIMO as complementary to the original signals. Consequently, we have 24 dimensional feature vectors for three MIMOs of an action in total. Additionally, we will make a feature selection process before the classification, and the details will be presented in Section 4.

### 3.5. Classification Method

The action recognition is a multi-classification problem. Here we adopt SVM (Support Vector Machine) to analyze data and recognize patterns [4]. In order to solve the nonlinear classification problem, we use a kernel function to map the input samples to high dimensional space, and then seek a maximum classification hyper plane in the high dimensional space for classification [23].

Let $X=\left\{x_{1},x_{2},\cdots ,x_{m}\right\}$ be the training set, and φ:X→H be the projection to the high dimensional space. So, the challenge is to solve the following quadratic programming problem:

$${}_{\omega ,\varepsilon ,\rho }^{min}\frac{1}{2}\|\omega^{2}\|+C\underset{i}{\sum }\varepsilon_{i}-\rho$$

Subject to

(5) $$\left(\omega \cdot \varphi \left(X_{i}\right)\right)\geq \rho -\varepsilon_{i},\varepsilon_{i}\geq 0$$

The discriminant function *F*(*x*) is in Equation (7).

(6) F(x)=sign(ω·φ(x)−ρ)

And we choose a quadratic polynomial kernel as the kernel function.

(7) $$K\left(x,y\right)={\left(<x,y>+1\right)}^{2}$$

## 4. Recognition and Evaluation

### 4.1. Training

Full-body action detection and recognition are useful in elderly care monitoring and anomaly detection. Among them, fall detection has a significant potential of usage in real applications and has been explored by both vision-based methods [24,25] and device-based (wearable sensor or smartphone) techniques [26,27] widely. We selected thirteen primitive actions listed in Table 1, and some of them are similar to falls. Furthermore, we can get more complex activities by combining two or more of these primitive actions.

**Table 1:** Thirteen selected primitive full-body actions in daily life.

No.	Descriptions	Action Key Points
AC1	Squat down to pick up something.	both legs do not bend.
AC2	Stand up from squatting state (AC1).	
AC3	Squat down to pick up something.	both legs bend.
AC4	Stand up from squatting state (AC3).	
AC5	Sit down on a chair.	
AC6	Stand up from a chair/bed.	
AC7	Lie down on the couch/bed.	from standing state
AC8	Sit up from lying state on the floor.	
AC9	Stand up from lying state on the couch/bed.	
AC10	Fall to the floor from standing up.	
AC11	Fall to the floor from sitting on a chair/bed.	
AC12	Stand up from floor from sitting state.	
AC13	Stand up from floor from lying state.	

In the training process, these actions were performed in an ordinary living room without household appliances on, including the lights and mobile phones. The transmitter and the receiving antennas were mounted on two separate identical wood desks at 1.2 m from the ground. At the receiver, an Intel Wi-Fi Link 5300 wireless NIC with three antennas and open source Linux wireless drivers [16] was applied to perform an envelope detection of the down-converted baseband signals. Five individuals performed the primitive actions ten times each at normal speeds in the middle of in-sight line-facing TP.

### 4.2. Feature Selection

**Figure 5:**
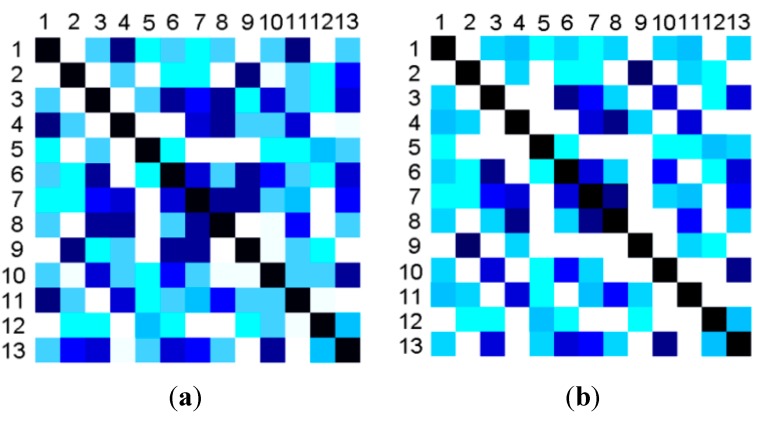
The comparison of similarity matrixes for 13 primitive actions between original features and selected features. The hue demonstrates the similarity between any two actions. (**a**) The similarity matrix for the original features; (**b**) The similarity matrix for the selected features.

As described in Section 3.3, we have 24 features for an action from the statistic data of each MIMO subplot, but they are set artificially and may have some redundancy, so a feature selection process was added. First of all, a forward selection process was performed by adding features one-by-one. The error rate of classification was taken as the evaluation function. Then, a backward selection step was performed by deleting the features one-by-one from the feature sets. The results of the feature selection were 14 features. Figure 5a demonstrates the similarities of the original 24 features for actions AC1 to AC13 and Figure 5b demonstrates the similarities of the selected 14 features. It is obvious that the selected features have more discriminative abilities. For the actions of different periods, DTW [22] was adopted and gave a similarity value instead of that of the feature PA (the period of an action).

### 4.3. Classification and Recognition

**Figure 6:**
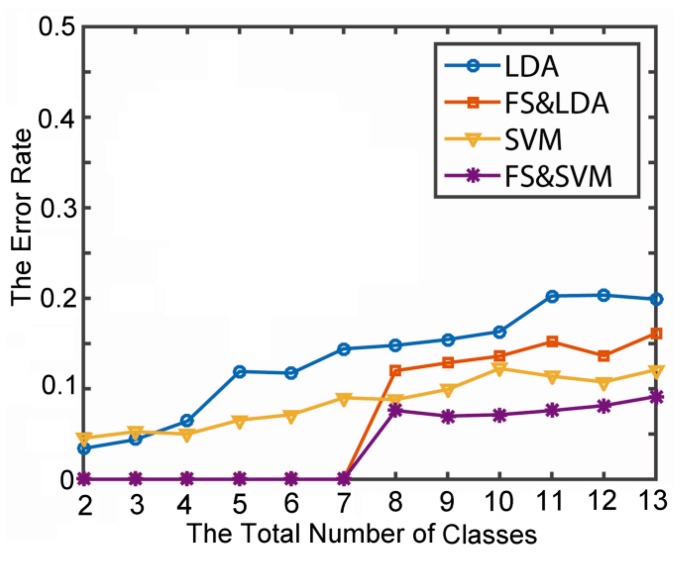
The average recognition error rate from cross-validation for 13 primitive actions.

In recognition of the solo in-place activities in Table 1, two multi-classification algorithms, Linear Discriminant Analysis (LDA) [28] and SVM with feature selection (FS), were evaluated and compared. The average error rate in cross-validation is shown in Figure 6. In this test, we randomly selected two instances from *N* (*N* = 1, …, 13) classes as the test dataset and took the average error rate as the result of the *N*th recognition error rate. The results showed that the SVM in combination with FS performs the best. Furthermore, with the action class number increases, the recognition rate decreases were relatively stable.

Another test for detecting falls using two-class SVM got a 95.2% recognition rate. In this experiment, we let five people of different body sizes each fall two times in random places and orientations at different speeds as a test set. Compared with the method in [11], our method can detect falls among six similar actions with a relatively high accuracy insensitive to the orientation and speed.

**Figure 7:**
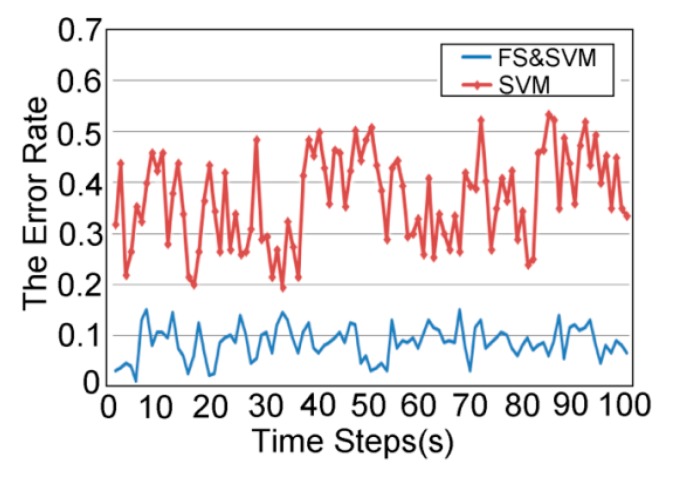
The average recognition error rate for actions in 100 (s).

In the last experiment, we let five people of different figures and ages perform actions including AC1–AC13 in 100 (s) time steps in any part of the room. Each primitive action was conducted at a normal speed (average 0.5 s–0.87 s) and separated by walking, standing, sitting, or another static state. The average error rates of recognition by SVM with and without feature selection are demonstrated in Figure 7. As the results show, the FS and SVM method has a lower error rate and can satisfy the requirement of real-time action recognition.

In practical applications, we have tested the robustness of our method. We put some household appliances including lights, television sets, electric rice-cookers, audio systems, and even mobile phones (in the standby mode or phone state) in a living room, and the results showed that they have very little disturbance on the filtered statistical CSIs computed by our method. Additionally, if we put the AP and TP at a proper height and position, the indoor furnishings also have little influence on CSIs. However, for more complex indoor environments (e.g., a factory workshop, a waiting room, and so on), we could exact the foreground CSI signals by Gaussian Mixture Model (GMM) [29,30] or other background subtraction methods which have been widely used in vision-based recognition.

## 5. Conclusions

Indoor wireless action recognition has spawned numerous applications in a wide range of living, production, commerce, and public services. The increase of mobile and pervasive computing has sharpened the need for accurate, robust, and off-the-shelf indoor action recognition schemes. In this paper, we explore the properties of CSIs of Wi-Fi signals and proposed a robust indoor daily human action recognition framework with only a pair of Wi-Fi transmission points and access points. We can achieve relatively high recognition accuracy for a set of similar daily actions insensitive to location, orientation, speed, and anthropometric differences. However, we cannot detect such small in-place actions (cooking, eating, playing video games, and so on) [13] because they have very small CSI fluctuations in our system settings.

Considering the advantages and limitations of wireless signals, we will put our effort into exploring how to gain more robust indoor action recognition rates in future work, and we think vision-based methods and Wi-Fi–based methods may be a good complement to each other in some specific circumstances.
